# Acceptability of an Intervention to Promote Viral Suppression and Serostatus Disclosure for Men Living with HIV in South Africa: Qualitative Findings

**DOI:** 10.1007/s10461-021-03278-w

**Published:** 2021-06-07

**Authors:** Mxolisi Mathenjwa, Hazar Khidir, Cecilia Milford, Nzwakie Mosery, Letitia Rambally Greener, Madeline C. Pratt, Kasey O’Neil, Abigail Harrison, David R. Bangsberg, Steven A. Safren, Jennifer A. Smit, Christina Psaros, Lynn T. Matthews

**Affiliations:** 1 MRU (MatCH Research Unit), Department of Obstetrics and Gynaecology, Faculty of Health Sciences, University of the Witwatersrand, Durban, South Africa; 2 Population Services International, South Africa, Johannesburg, South Africa; 3 Harvard Medical School, Boston, MA USA; 4 Massachusetts General Hospital, Boston, MA USA; 5 Brown University School of Public Health, Providence, RI USA; 6 Oregon Health Science University, Portland, OR USA; 7 University of Miami, Miami, FL USA; 8 University of Alabama at Birmingham (UAB), Division of Infectious Disease, Birmingham, Alabama USA

**Keywords:** South Africa, Men living with HIV, Safer conception, Treatment as prevention, HIV-serostatus disclosure

## Abstract

Men living with HIV (MLWH) often have reproductive goals that can increase HIV-transmission risks to their pregnancy partners. We developed a safer conception intervention for MLWH in South Africa employing cognitive behavioral skills to promote serostatus disclosure, ART uptake, and viral suppression. MLWH were recruited from an HIV clinic near Durban, South Africa, and encouraged to include partners in follow-up visits. Exit in-depth interviews were conducted with eleven men and one female partner. The emerging over-arching theme is that safer conception care mitigates internalized and community-level HIV-stigma among MLWH. Additional related sub-themes include: (1) safer conception care acceptability is high but structural barriers challenge participation; (2) communication skills trainings helped overcome barriers to disclose serostatus; (3) feasibility and perceived effectiveness of strategies informed safer conception method selection. Our findings suggest that offering safer conception care to MLWH is a novel stigma-reducing strategy for motivating HIV prevention and treatment and serostatus disclosure to partners.

## Introduction

South Africa has the largest population of people living with HIV (PLWH) in the world [1]. More than 30% of PLWH in South Africa are in stable serodifferent relationships, or in a sexual partnership with a partner at high risk for acquiring HIV [2, 3]. In South Africa, only an estimated 78% of men living with HIV (MLWH) know their serostatus, of these, 52% access antiretroviral therapy (ART), and 42% are virally suppressed [4]. Because MLWH have fertility desires, goals and intentions just as HIV-negative individuals do, serodifferent couples may risk HIV transmission to achieve pregnancy [5–7]. While the exact contribution of periconception HIV transmission is not known, women face increased risks of HIV acquisition during early pregnancy, which overlaps with periconception periods [8]. In addition, growing recognition of reproductive rights for PLWH, advances in HIV prevention that align safer conception care with broader HIV treatment and prevention goals, and improvements in access to HIV care resulting in longer life expectancies make reproductive health care integration into HIV care a growing priority [9, 10].

Established guidelines outline safer conception strategies including ART-mediated HIV-RNA suppression for the partner living with HIV, pre-exposure prophylaxis (PrEP) for the uninfected partner, condomless sex timed to ovulation, and semen processing technologies [10–13]. Data suggest that implementation of counselling on these strategies reduces HIV transmission to near zero [14, 15]. However, healthcare workers lack basic training and system-level support to counsel clients, so most PLWH do not receive safer conception care [16–21].

Men in South Africa and from many societies inform decisions around contraception and HIV prevention [22, 23]. Verticalization of care and reproductive health services, which traditionally reach women, limit how and whether men access care and contribute to HIV- and reproductive health-related stigma and discrimination for men [24]. Men often express reproductive goals [25, 26] but are less likely to access HIV testing and treatment, and are more likely to be lost to follow-up relative to women [27–34]. Given gender norms about reproductive roles and stigma towards PLWH having children, MLWH are the least likely to be offered reproductive health counselling [34–39, 40]. As the HIV and reproductive health community urge greater engagement from men [24, 35–39], supporting men to safely meet important personal and sociocultural reproductive goals may provide an opportunity to increase demand for services and thus support increases in HIV testing, engagement in care, uptake of ART, and HIV RNA suppression. Providing safer conception services to men also establishes an opportunity for disclosure and to link women to HIV prevention and treatment opportunities.

Prior work demonstrates that MLWH express interest in counselling that helps them safely meet reproductive goals and are motivated to change risk behavior to protect their baby from HIV acquisition [40–45]. Existing safer conception interventions in sub-Saharan Africa have worked with couples [21]. In testing a safer conception counselling and service intervention for couples affected by HIV (with at least one partner testing positive for HIV), researchers in Johannesburg demonstrated that their intervention could help meet the reproductive goals of serodifferent couples while minimizing HIV transmission. However, while the men who attended visits valued the service, the team faced difficulties engaging male partners [46, 47]. In a safer conception study in Kenya, there was high uptake of safer conception strategies and zero HIV transmission events among HIV-serodifferent couples [14].

How to effectively engage men who are not yet in mutually disclosed partnerships into safer conception care remains uncertain. We developed a male-focused, manualized, cognitive behavioral therapy (CBT)-based intervention to encourage and support MLWH who want to have children with HIV-exposed partners to adopt safer conception behaviors, including HIV-serostatus disclosure and initiation of ART. Men were encouraged to include their desired pregnancy partners in the intervention. Here we report on exit qualitative In-Depth Interviews (IDIs) conducted with men and a pregnancy partner who participated in a safer conception intervention in KwaZulu-Natal, South Africa between 2015 and 2017. The aim of this analysis was to explore successes and challenges of the safer conception intervention for MLWH in South Africa.

## Methods

### The Safer Conception Intervention

**Fig. 1:**
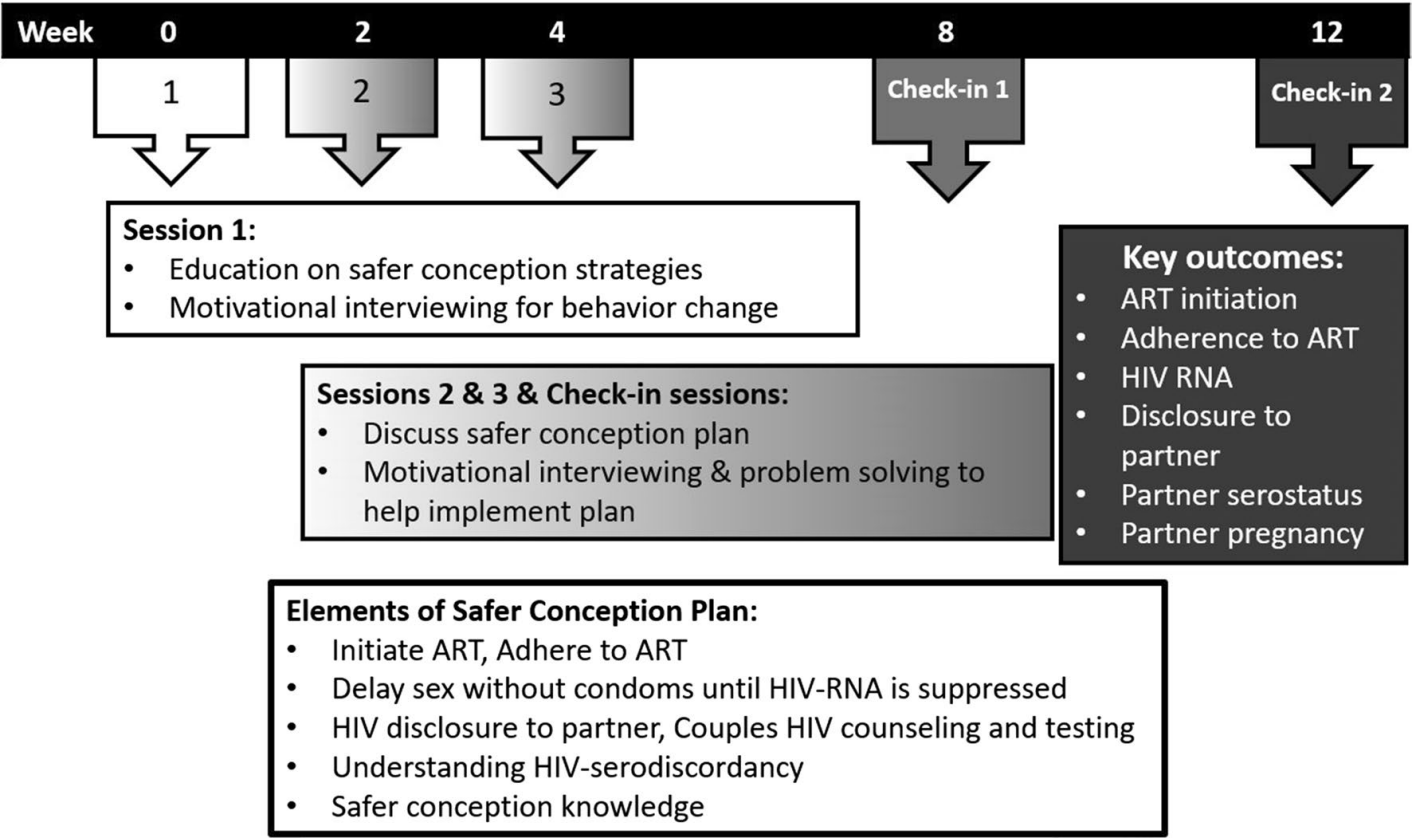
Schematic of intervention sessions

We developed a safer conception intervention, *Sinikithemba Kwabesilisa,* meaning ‘Helping Men Have Healthy Babies’ in isiZulu, informed by prior formative work, intervention development is described in a prior publication [45]. The final intervention (Fig. 1) consisted of five safer conception counselling sessions employing cognitive behavioral skills including problem solving and motivational interviewing in three sessions over 12 weeks plus two check-in sessions. One-on-one safer conception counselling offered HIV and safer conception education, assistance creating a safer conception plan (referred to as a Healthy Baby Plan), and communication and problem-solving skills for implementing their desired safer conception plan. Participants’ motivation to make behavioral changes was also explored. Sessions promoted ART uptake and adherence, delay of condom-less sex until suppression of HIV-RNA, timing condom-less sex to peak fertility, and HIV-serostatus disclosure (supported by role play and communication skills building). PrEP was not available in South Africa at the time this study was conducted.

### Setting and Participants

We recruited men from a comprehensive HIV and STI clinic in a township near Durban, South Africa to participate in the intervention. Eligible men were 20–45 years old, had known their HIV-positive status for at least 6 months, were ART naïve or accessing ART for less than 3 months, and were planning to have a baby in the next year with an HIV-negative or serostatus-unknown partner. Men were encouraged to invite their desired pregnancy partners to follow-up counseling sessions. Their female partners were eligible if they were 18 years or older in age, and partnered with an enrolled man, confirmed through a partnership verification questionnaire at enrollment [48]. Consenting, enrolled pregnancy partners completed pregnancy and HIV testing and a baseline questionnaire. All were eligible for IDIs.

### Data Collection and Analysis

**Fig. 2:**
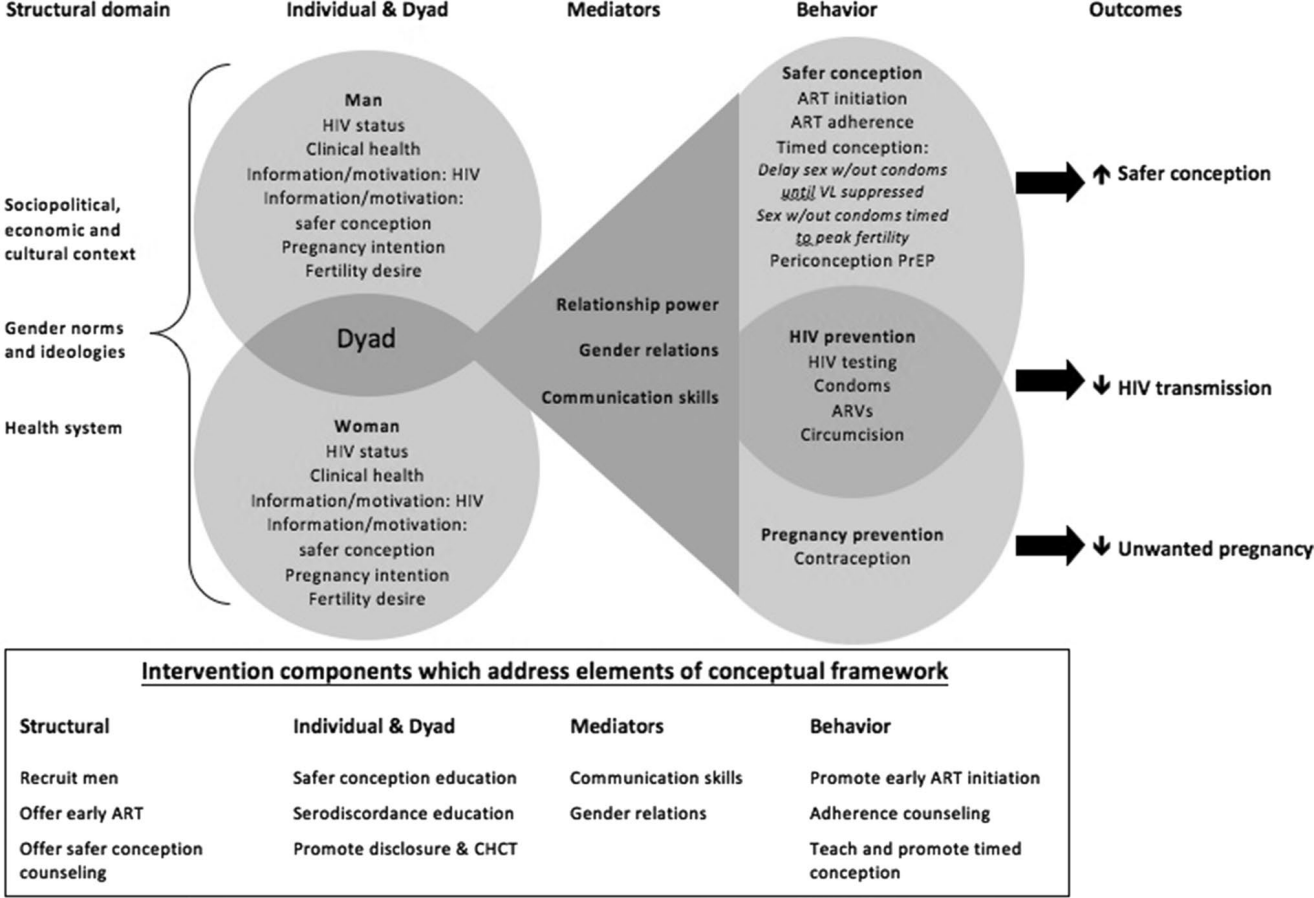
Safer conception conceptual framework (1) & key elements addressed by the intervention

Exit IDIs were conducted separately with eleven men and one female partner in a private room in a one-on-one setting in either *isiZulu* or English, depending on the participant’s choice, by a gender-concordant facilitator fluent in both languages. Eligibility for IDI participation was based on completing the initial five sessions for men (88% of enrolled men completed the sessions), and at least one session for female participants. Semi-structured exit IDI guides explored intervention delivery logistics, intervention content, female partner participation, HIV-serostatus disclosure, and a participant-centered evaluation of their participation. Interviews lasted approximately 60–90 min. Three female partners participated in the intervention with only one electing to complete the exit IDI. Interviews were audio-recorded, transcribed when indicated and translated into English. Transcripts were reviewed by several members of the research team (MM, LTM, HK, LG) to identify categories and themes informed by an ecological conceptual framework [49] (Fig. 2). We iteratively-developed a codebook to organize text into coding categories using thematic analysis. MM coded transcripts with oversight from LTM and CM using NVIVO v.10 software (QSR International). Based on the coding the coding team discussed emergent themes with the larger research team to ensure consensus.

## Results

### Demographic Characteristics

Eleven men and one female pregnancy partner completed IDIs at study exit. Table 1 presents demographic characteristics of the participants who completed IDIs. Men were 25–35 years old, all reported living with HIV for at least a month, and all were in a stable sexual relationship with a potential pregnancy partner for at least 6 months**.** Seven men reported an HIV-uninfected female partner, and three did not know their potential pregnancy partners’ HIV-serostatus. One man reported his pregnancy partner to be living with HIV after enrolment.

**Table 1:** Enrolment characteristics of exit in-depth interview participants

	Men (N = 11) Median (Range) N (%)	Woman (N = 1)
Age (years)	30 (25–35)	25
Education		
Completed secondary school	6 (55%)	
Completed tertiary school	1 (9%)	1
Full-time employment	5 (45%)	1
HIV-serostatus disclosed to pregnancy partner	5 (45%)	NA
CD4 cell count (cells/mm3)	598 (165–959)	NA
Pregnancy partner type	100% long-term girlfriend	NA
Pregnancy partner HIV-serostatus		NA
Negative	7 (64%)	
Unknown	3 (27%)	
Positive	1 (9%)	

Additionally, a 25 year-old female pregnancy partner participated in an IDI. She had completed a tertiary qualification (or had completed some university studies), was full-time employed, and had an unknown HIV serostatus at enrolment.

### Emergent Themes

Guided by our conceptual framework (Fig. 2), the intervention was designed to promote safer conception behaviors (ART uptake, ART adherence, viral load suppression (VLS), serostatus disclosure) through improving safer conception knowledge, communication skills, and gender relations. We anticipated that demand would be high, and men would overcome structural barriers to participate in a clinic-based intervention. The over-arching theme that emerged from the data was that safer conception care may mitigate internalized and community-level HIV stigma experienced by MLWH in the context of living with HIV and wanting to have children. Sub-themes which also reflect challenges with stigma in this context include high acceptability for safer conception care but structural barriers to participate in a clinic-based intervention are not easily overcome; communication skills trainings were pivotal to overcoming individual and dyadic-level barriers to participate in safer conception care, client perceptions of feasibility and effectiveness informed safer conception method selection.

#### Safer Conception Care may Mitigate HIV Stigma Among MLWH

Men reported that community assumptions that MLWH could not have children reduced their sense of value in society and contributed to ongoing internalized stigma. Some of the men had lost hope of having HIV-uninfected children; after participating in sessions, they reported that the safer conception information had motivated and renewed their hopes of “normal” and healthy lives for themselves and their future children.

“It [safer conception intervention] helped me a lot. Basically, I had lost hope that I could ever have a child. … but now this has changed my mind that even if I am HIV positive, I can still have uninfected children using these strategies that we had talked about. I now feel like my HIV status does not change the way I need to live, I can still live … […] I can still do whatever that I had wanted to do.”—M1007, 35 years, male.

“I think it had a good impact, it helped me a lot. Psychologically, I was not able to even live and feel like a person… What came through my mind was that I will not by any chance have a baby, so it means I will just die in pain. All those thoughts have been washed away. If I can just take ART as prescribed …” – M1002, 31 years, male.

Men also suggested that community messaging to understand options for PLWH having children without HIV transmission was important to address stigma and to disseminate information to other PLWH.

“… this [programming] should not only be for the people that have been infected but even the uninfected ones. It would help so that everyone gets to know about this because you might not have the virus today, but one day you might find yourself infected, so that would help …”—M1003, 29 years, male.

Furthermore, they endorsed the one-on-one sessions, which enabled them to discuss health-related matters as well as concerns about living with HIV and childbearing without feeling stigmatized. They also articulated that the intervention could be strengthened by including peer support.

“I was able to ask with ease because it was a one-on-one session and if there were many people, I would have probably been scared to ask because of the people who would have been there, it really helped me a lot … I think that [group session] as well would have its own advantages because I think people are not the same, their behavior is also not the same, maybe if you hear from another person about how he does things and what was problematic for him, then it would have helped in a way that you would also not end up in the same problem.”—M1002, 31 years, male.

#### Acceptability of Safer Conception Care is High but Structural Barriers to Participate in a Clinic-Based Intervention During Working Hours are not Easily Overcome

The intervention specifically sought to reach men of reproductive ages [20–45 years]. Contextually, this group (and their partners) were studying, working, or seeking employment, which is reflected in our recruitment challenges. Intervention participants spoke to the challenges of scheduling sessions during routine clinic hours (weekdays 8a.m.–2 p.m.). Men acknowledged that the intervention required an investment of their time, which they were motivated to do. While they were able to negotiate time from social activities, they had less flexibility negotiating time away from work.

“What I can say, my brother, is that continue educating people in the way that you have done with me because I know it is not easy getting people because this programme requires time and people are employed …”—M1012, 33 years, male.

“… because sometimes others would have a problem coming through because of transport fare, employment. They can only request some time out when they have to get their medications, then they would get to do the whole thing in one day.”—M1010, 28 years, male.

Only three men involved their partners in the sessions. Men discussed how accessing the clinic during working hours and structural factors (including that partners often do not cohabitate) impacted the ability of women to attend clinic with them.

“She wished [to attend], the thing is that she had recently found a new job, so that is the most concrete reason, because she had recently changed jobs. If it was her old job, perhaps she could have been able to request some time out but then for this new job, she was still not yet at a position where she could ask for some time.”—M1013, 30 years, male.

While attending the clinic was challenging, men highlighted the importance of repeated sessions given the time required to build rapport and trust.

“… the crisis that we are facing is us, as males, we are afraid of coming out into the open about things, we end up not talking about things. […] you would start picking up on the third or fourth sessions… and that is when you start opening up and realising that these [counsellors] are good people. […] because we are the kind of people who do not like to be sympathized for …”—M1007, 35 years, male.

#### Communication Skills Trainings were Pivotal to Overcoming Individual and Dyadic-level Barriers to Participate in Safer Conception Care, Including Disclosure

We designed this study to recruit men without requiring, but encouraging, partner involvement. After the counselling sessions most participants realized that implementation of many safer conception strategies required involving their partners.

“… because it takes two to tango, so you can’t have one person in the relationship to be the one [who participates in the counselling] because that basically means that he/she will be the one who will be calling the shots … without you even knowing about it. It is better if you would both know.”—M1007F, 25 years, female.

“[The best approach] is working hand in hand with your partner because it is not about one person, it is about two people. Secondly it is making sure that you take your medication accordingly because they are the key, without medication this will never be done, [or] it would be done, but you would be able to infect your partner. Thirdly is disclosing …”—M1003, 29 years, male.

While men appreciated the value of serostatus disclosure, challenges including fear of relationship dissolution were common.

“What I can tell you, my brother, is that the main problem that we are facing right now is that we hide things from each other. That is the most common problem that we are facing and, my brother, disclosing to your partner, the first thing that runs through your mind is that she will run …”—M1012, 33 years, male.

This intervention introduced communication skills training and support to help men disclose.

“… I disclosed after I was in the study. I had thought about disclosing, but I did not know when I was going to disclose. But if the study was not existing, I do not think I would have disclosed sooner like I did.”—M1010, 28 years, male.

“I did not want to disclose in any random way, I wanted to disclose to her in a way that I could also be able to comfort her after finding out and should be able to talk to her thereafter and to be able to make her feel that there is still life, and life is continuing. So, your programme helped me a lot and to sharpen me in terms of strategies of disclosing to your partner.”—M1007, 35 years, male.

The female partner also shared the need for support to disclose. She discussed couples-based counselling and testing as an important strategy and alluded to her experience:

“I do not recommend that you go test alone, because if you go get tested alone, chances of you disclosing the right information to your partner are very slim. I am talking from experience. It is better if you go there together…”—M1007F, 25 years, female.

The female participant described that her partner’s regular interactions with providers enhanced HIV and safer conception knowledge within their relationship.

“It [the counselling program] had a positive impact on our relationship because that is when I got to notice how serious he is about things and how to go about doing things the right way.”—M1007F, 25 years, female.

#### Method Choice is Influenced by Preference, Feasibility, and Perceptions of Efficacy

Feasibility, cost, and perceptions of efficacy led most men to choose treatment as prevention and condomless sex timed to peak fertility as their safer conception strategies.

Men described that counselling about Undetectable = Untransmittable (U = U) and the power of ART on morbidity and mortality inspired hope for their future, independent of plans for building families.

“There is so much that I have learnt, my brother., … such as taking treatment which is what I wanted … I need to take treatment and suppress the virus so that my partner can be alright and protect her from being infected.”—M1005, 25 years, male.

For some men, problem solving to create adherence support helped them adhere to daily medication and accomplish the goal of treatment as prevention as a safer conception strategy.

“Firstly, because if you say taking medication accordingly, … taking medication accordingly does not only stop there, it bases itself on you getting a person who will support you or have something that will encourage you to take treatment. Because if [you] only say ‘take medication accordingly’ I would also say that and just leave and spend time with my friends and so forth and perhaps forget. When we meet again I would ask; are you taking them accordingly? And then you would [say] ‘yes’, but then there has to be something that is there that would remind you even if you are on the other side and remember that okay it is time. My own was that me and my partner used to chat on WhatsApp and then she would perhaps remember that it is 9 p.m. then she would tell me ‘hey it is time, take that thing’.”—M1013, 30 years, male.

Timing sex to peak fertility was described as desirable because it was a “natural” process.

“Well my brother I prefer timed sex … But I do have fears, perhaps I have to say that clearly, I do have those fears because I would not like my baby to … should I have the baby and found herself/himself in the same status as I am but I prefer the timed sex. Firstly, I believe in what is natural (timed sex), maybe that is the main reason, so if I were to have a baby in another way I do not think I would feel well … it is just that I am not used to them (the unchosen strategies), and secondly things that are technical and electronic do not make me think I would be fine with that.”—M1007, 35 years, male.

“And then I chose to have a baby through timed sex, yes I loved that situation because it is something that happens and they know that at this particular time, it is known that the female has no problem and can actually conceive and I see this as an easy strategy because it is part of life that there is a time where men and women have to meet.”—M1008, 34 years, male.

Semen processing was not a preferred strategy as it was perceived to be costly and men were concerned about the low likelihood of successful pregnancy and uncertainty of paternity. Men described a lack of trust and feelings that this was an over-medicalized procedure to conceive (which is traditionally a private experience). The closer to ‘normal’ the strategy the greater comfort and sense of feasibility was expressed.

“…it is said to be costly, that is number one, number two it is not hundred percent successful because it first has to start at laboratories for the semen to be washed, it would arrive in the female when it is weak, you see that… as it is going to be washed it will require money, get to doctors who also require money and at the end that would not be a success.”—M1003, 28 years, male.

“… Sperm washing is very much problematic because a lot of things can happen during the whole process because we would not be sure that it is really my sperm. Because there are surely many sperms that are being processed there. If there be a mistake and that then sperms get mistakenly swapped.”—M1015, 29 years, male.

## Discussion

We describe exit interview data from a male-centered safer conception pilot study conducted in an HIV-endemic setting in Durban, South Africa. Men who completed a 12-week safer conception intervention to promote ART uptake, couples’ communication, and HIV-RNA suppression described the importance of this intervention at community- and individual-levels, given their desires to have children and the lack of information available to them about how to safely meet reproductive goals. Men highlighted how internalized- and community-level HIV stigma and community- and provider-stigma towards PLWH having children make it difficult for men to discuss their serostatus or reproductive plans. While it was challenging to overcome structural barriers to attend clinic-visit-linked intervention sessions, men felt the number of sessions were necessary due to the time required to develop rapport and trust the interventionists. The challenges of HIV-serostatus disclosure to partners were highlighted and participants articulated the value of discussing their fears and challenges and opportunities with a supportive counsellor who had time to listen and follow-up, and skills to improve partnership communication. Men chose safer conception methods that were cost-free (to them) and aligned with larger HIV treatment goals such as ART-mediated viral suppression. We are unaware of other programs focusing on safer conception for men as individuals—but the literature highlights the need for this work [40, 50, 51] and our findings suggest that offering safer conception care to MLWH who want a child is novel stigma-reducing strategy for motivating HIV prevention and treatment, serostatus disclosure to partner, and addressing childbearing stigma among MLWH.

Patriarchal gender ideologies and hegemonic notions of masculinity limit opportunities for men to engage in healthcare [52,53]. Barriers to HIV testing and prevention for men include discomfort with the health care system, lack of health education, limited service availability conflicting with work schedules, and the limitations of patient-provider discourse in offering or explaining the purpose and reasons behind certain services [54, 55], reflected as structural barriers in our conceptual framework (Fig. 2). We developed a client-centered intervention with flexible scheduling (although we were limited to clinic working hours), and offered one-on-one counseling provided by local men trained to offer supportive care. While men are eager to have HIV-uninfected children, they do not receive reproductive counseling to achieve this goal [41, 50, 56, 57, 58, 59]. Harnessing masculine norms that promote responsibility for building and supporting healthy families may increase men’s HIV care engagement [50, 52, 53, 58, 60, 61]. We leveraged these cultural and gender norms [59] to develop care centered on men’s needs. Brief communication skills trainings were able to influence communication based challenges (often rooted in gender based norms around how men and women should communicate) associated with the execution of a safer conception plan. This provided tools for men to disclose their status in many cases, and allow the female partner to participate in the safer conception plan. Lastly, our data reflect that men’s preferences for safer conception fell under the domain of HIV prevention as reflected in our conceptual framework, as they were the most acceptable, easiest to implement, and were perceived to have the greatest efficacy.

Men who accessed our program seemed to thrive in this patient-centered care model, describing an optimistic outlook on the future and motivation to employ techniques to prevent HIV transmission and improve their own health. While some community programs for men have been successful [36, 60, 62], we are not aware of other clinic-based programs that have provided male-centered programming in South Africa. One facility-based intervention to promote medical male circumcision (MMC) in Zambia promoted training for providers and male-focused educational sessions and was successful in increasing uptake of MMC [63], suggesting that facility-based interventions do have the potential to improve care. In Leostho, a male-centered HIV clinic program run by male providers has demonstrated high-demand and reach [64]. Gender-specific care delivered by community counsellors has been successful with the mentor mothers program to promote antenatal care and reduce perinatal transmission in South Africa and other settings in sub-Saharan Africa [65].

MLWH who want to have children with an uninfected partner can be affected by multiple stigmas including internalized, community-level, and provider stigmas associated with having HIV, having children while living with HIV, and being in a sero-different partnership [66, 67]. Stigma and discrimination compromise the health and safety of PLWH and their partners through decreased social and emotional support, decreased access to unbiased healthcare, and difficulties utilizing societal resources [68]. Partners of PLWH report being pressured to leave their relationships, or they are considered “positive by association” [68]. HIV-affected couples are often discouraged from having children, with stigma and fear of HIV-transmission between partners and from mother to child as the most prominent barriers [69, 70]. While we did not design this as a stigma reducing intervention, Helping Men Have Healthy Babies’ participants described that the program addressed some forms of internalized stigma by making participants feel like they were able to safely have families. We believe that the intervention impacted men’s experience of internalized and provider-level stigma, represented in the structural domain of our framework (Fig. 2), through counselling and education about U = U messages (individual), offering strategies to prevent HIV-transmission to uninfected partners (individual), and also by normalizing wanting to have children and offering options and supportive, nonjudgmental attitudes in the context of a healthcare setting (provider). The sessions in this intervention were one-on-one. However, men articulated that group sessions would also be helpful. A blended approach of one-on-one sessions with group sessions for those who are able to share may be ideal. Reflecting and problem solving could be facilitated by understanding how others have coped but also normalizes the challenges faced. There are very few avenues for men to discuss their fertility desires, difficulties disclosing and without disclosure they are unable to seek support when needed. This intervention gave them this much needed support and provided a space that normalized living with HIV. Future safer conception work with this population should explore the role of multi-level stigmas as a moderator of key outcomes of HIV viral suppression and serostatus disclosure [71, 72].

Engaging men in safer conception care through women partners has proven difficult in multiple settings, with most safer conception programs recruiting fewer than 60% of the male partners of female participants [15, 46, 73], yet men are critical to many periconception decisions and behaviors [74]. We enrolled men who had not necessarily disclosed to their pregnancy partners and aimed to promote serostatus disclosure through communications trainings and through counselling about the importance of knowing partner serostatus to inform safer conception care. Men suggested that the supportive environment, working through fears using problem solving and motivational interviewing, and having time to work through their desired actions at their own pace gave them the confidence and techniques to disclose to their partners. Larger scale work will be required to understand how effective our intervention is at motivating disclosure compared to standard of care, but these preliminary findings suggests that this may be an acceptable approach for those who are not yet ready to engage in couples-based strategies.

Given the limits on everyone’s resources (clients, providers, health systems), these data highlight the importance of prioritizing safer conception methods that are cost-free to patients and align with broader HIV treatment and prevention goals. Placing ART-mediated viral suppression and testing of and disclosure to partners aligns with 95-95-95 UNAIDS goals [75] and simplifies opportunities for integrating comprehensive reproductive health care into HIV treatment and prevention programs. As PrEP becomes increasingly available, PrEP for uninfected partners may also be a key component [76, 77]. Semen processing, timing sex to peak fertility, and artificial insemination are more nuanced approaches that are unique to safer conception care and do not align with broader treatment and prevention goals and may be harder to integrate into existing services without substantial added value for those without baseline infertility.

Importantly while those who participated in interviews reported demand for and acceptability of the intervention, our findings highlight how challenging it is for people of reproductive age, who are otherwise healthy, to access this care through additional clinic visits when they are busy studying, working, or seeking employment [31, 34–38, 64, 78]. Thus, it is important for future programming to consider working outside of the traditional clinic setting, possibly moving into communities with outreach and community mobilization efforts or using mHealth approaches [79, 80] to address other structural factors that limit HIV-care engagement for men [43].

Finally, only men who completed our intervention were eligible for IDIs—thus we are lacking interview feedback from men who chose not to or were otherwise unable to complete the intervention. There were few IDIs, but men shared data aligning with findings from other work [44, 67, 68]. Given the enthusiasm for engaging men in HIV care and reproductive health and the scarcity of data on how to achieve this, these limited data remain important to inform future studies [78, 81].

## Conclusions

Our data suggest that MLWH who choose to have children and participate in this intervention will engage in care to address their treatment needs and protect partners and infants from HIV infection. Current data support integration of messages about the safety of having children when both partners know their status and those living with HIV are virally suppressed. Disseminating these messages at the community level may support PWH to meet reproductive goals while ameliorating stigma at the provider and patient level. Given the multi-level stigmas that this population experiences in accessing health care and the minimal community-knowledge about safer conception options, multiple sessions may be required to communicate messages and convey support for men and promote behaviors that are complex (e.g. HIV-serostatus disclosure). Ongoing support may be required for men whose reproductive goals shift and may not come to fruition during a brief intervention. Future iterations of this research will require community engagement to reach men and couples who are not already accessing HIV care and explore how existing health care systems can be leveraged to provide support. Future studies should compare the efficacy of individualized counselling compared to standard of care and explore the mediating effects on stigma on serostatus disclosure, viral suppression, and HIV serostatus of partner.

## Data Availability

Due to the limits of the consent, the small sample size, and nature of qualitative data collected, we are unable to make the data available in a repository. We are able to review data access requests for elements of raw data. Requests may be sent to the UAB Center for Clinical and Translational Science via CCTS@uab.edu; primary study authors may also be contacted.

## Code Availability

NA.

## References

[1] UNAIDS. Global AIDS update 2019—Communities at the centre, 2019. http://rstesa.unaids.org/publications/global-publications/2019/item/209-global-aids-update-2019-communities-at-the-centre.

[2] Lingappa JR, Lambdin B, Bukusi EA, Ngure K, Kavuma L, Inambao M (2008). Regional differences in prevalence of HIV-1 discordance in Africa and enrollment of HIV-1 discordant couples into an HIV-1 prevention trial. PLoS ONE.

[3] Kilembe W, Wall KM, Mokgoro M, Mwaanga A, Dissen E, Kamusoko M (2015). Knowledge of HIV serodiscordance, transmission, and prevention among couples in Durban, South Africa. PLoS ONE.

[4] Human Sciences Research Council (HSRC). The Fifth South African National HIV prevalence, incidence, behaviour and communication survey, 2017: HIV impact assessment summary report. 2018.

[5] Mmbaga EJ, Leyna GH, Ezekiel MJ, Kakoko DC (2013). Fertility desire and intention of people living with HIV/AIDS in Tanzania: A call for restructuring care and treatment services. BMC Public Health.

[6] Mindry DL, Crankshaw TL, Maharaj P, Munthree C, Letsoalo T, Milford C (2015). "We have to try and have this child before it is too late": Missed opportunities in client-provider communication on reproductive intentions of people living with HIV. AIDS Care.

[7] Aepfelbacher JA, Chaudhury CS, Mee T, Purdy JB, Hawkins K, Curl KA (2020). Reproductive and sexual health knowledge, experiences, and milestones in young adults with life-long HIV. AIDS Care.

[8] Thomson KA, Hughes J, Baeten JM, John-Stewart G, Celum C, Cohen CR (2018). Increased risk of female HIV-1 acquisition throughout pregnancy and postpartum: A prospective per-coital act analysis among women with HIV-1 infected partners. J Infect Dis.

[9] Gruskin S, Ferguson L, O'Malley J (2007). Ensuring sexual and reproductive health for people living with HIV: An overview of key human rights, policy and health systems issues. Reprod Health Matters.

[10] Matthews LT, Mukherjee JS (2009). Strategies for harm reduction among HIV-affected couples who want to conceive. AIDS Behav.

[11] Bekker LG (2011). Guideline on safer conception in fertile HIV-infected individuals and couples. South Afr J HIV Med.

[12] Matthews LT, Smit JA, Cu-Uvin S, Cohan D (2012). Antiretrovirals and safer conception for HIV-serodiscordant couples. Curr Opin HIV AIDS.

[13] Davies N (2018). Guidelines to support HIV-affected individuals and couples to achieve pregnancy safely: Update 2018. South Afr J HIV Med.

[14] Heffron R, Ngure K, Velloza J, Kiptinness C, Quame-Amalgo J, Oluch L (2019). Implementation of a comprehensive safer conception intervention for HIV-serodiscordant couples in Kenya: Uptake, use and effectiveness. J Int AIDS Soc.

[15] Schwartz SR, Bassett J, Mutunga L, Yende N, Mudavanhu M, Phofa R (2019). HIV incidence, pregnancy, and implementation outcomes from the Sakh'umndeni safer conception project in South Africa: A prospective cohort study. Lancet HIV.

[16] Goggin K, Mindry D, Beyeza-Kashesya J, Finocchario-Kessler S, Wanyenze R, Nabiryo C (2014). "Our hands are tied up": Current state of safer conception services suggests the need for an integrated care model. Health Care Women Int.

[17] Steiner RJ, Dariotis JK, Anderson JR, Finocchario-Kessler S (2013). Preconception care for people living with HIV: Recommendations for advancing implementation. AIDS (London, England).

[18] Black V, Davies N, Williams BG, Rees HV, Schwartz SR (2016). Establishing conception intentions and safer conception services for eliminating the vertical, and reducing the horizontal, transmission of HIV. BJOG.

[19] Steiner RJ, Black V, Rees H, Schwartz SR (2016). Low receipt and uptake of safer conception messages in routine HIV care: Findings from a prospective cohort of women living with HIV in South Africa. J Acquir Immune Defic Syndr.

[20] West N, Schwartz S, Phofa R, Yende N, Bassett J, Sanne I (2016). "I don't know if this is right … but this is what I'm offering": healthcare provider knowledge, practice, and attitudes towards safer conception for HIV-affected couples in the context of Southern African guidelines. AIDS Care.

[21] Joseph Davey D, West S, Umutoni V, Taleghani S, Klausner H, Farley E (2018). A systematic review of the current status of safer conception strategies for HIV affected heterosexual couples in Sub-Saharan Africa. AIDS Behav.

[22] Nattabi B, Li J, Thompson SC, Orach CG, Earnest J (2009). A systematic review of factors influencing fertility desires and intentions among people living with HIV/AIDS: Implications for policy and service delivery. AIDS Behav.

[23] Wegner MN (1998). Men as Partners in Reproductive Health: From Issues to Action. Intern Perspect Sex Reprod Health.

[24] Ramirez-Ferrero E, Lusti-Narasimhan M (2012). The role of men as partners and fathers in the prevention of mother-to-child transmission of HIV and in the promotion of sexual and reproductive health. Reprod Health Matters.

[25] RHRU, Population Council, Frontiers in Reproductive Health Program, International FH. Involving men in maternity care in South Africa. USAID HRN-A-00-98-00012-00 and population council subagreement AI199.43A; 2004.

[26] Cooper D, Moodley J, Zweigenthal V, Bekker LG, Shah I, Myer L (2009). Fertility intentions and reproductive health care needs of people living with HIV in Cape Town, South Africa: Implications for integrating reproductive health and HIV care services. AIDS Behav.

[27] Osler M, Hilderbrand K, Goemaere E, Ford N, Smith M, Meintjes G (2018). The continuing burden of advanced HIV disease over 10 years of increasing antiretroviral therapy coverage in South Africa. Clin Infect Dis.

[28] Mitchell S, Cockcroft A, Lamothe G, Andersson N (2010). Equity in HIV testing: Evidence from a cross-sectional study in ten Southern African countries. BMC Int Health Hum Rights.

[29] Camlin CS, Ssemmondo E, Chamie G, El Ayadi AM, Kwarisiima D, Sang N (2016). Men "missing" from population-based HIV testing: Insights from qualitative research. AIDS Care.

[30] Dovel K, Yeatman S, Watkins S, Poulin M (2015). Men’s heightened risk of AIDS-related death: The legacy of gendered HIV testing and treatment strategies. AIDS (London, England).

[31] Mambanga P, Sirwali RN, Tshitangano T (2016). Factors contributing to men’s reluctance to seek HIV counselling and testing at primary health care facilities in Vhembe district of South Africa. Afr J Prim Health Care Fam Med.

[32] Centers for Disease Control and Prevention (CDC) (2013). Differences between HIV-Infected men and women in antiretroviral therapy outcomes - six African countries, 2004–2012. MMWR Morb Mortal Wkly Rep.

[33] Giles ML, Achhra AC, Abraham AG, Haas AD, Gill MJ, Lee MP (2018). Sex-based differences in antiretroviral therapy initiation, switching and treatment interruptions: Global overview from the International Epidemiologic Databases to Evaluate AIDS (IeDEA). J Int AIDS Soc.

[34] Treves-Kagan S, El Ayadi AM, Pettifor A, MacPhail C, Twine R, Maman S (2017). Gender, HIV testing and stigma: The association of HIV testing behaviors and community-level and individual-level stigma in rural South Africa differ for men and women. AIDS Behav.

[35] Mbizvo MT, Bassett MT (1996). Reproductive health and AIDS prevention in sub-Saharan Africa: The case for increased male participation. Health Policy Plan.

[36] Peacock D, Stemple L, Sawires S, Coates TJ (2009). Men, HIV/AIDS, and human rights. J Acquir Immune Defic Syndr.

[37] Campbell CA (1995). Male gender roles and sexuality: implications for women’s AIDS risk and prevention. Soc Sci Med.

[38] The International Conference on Population and Development, Cairo Egypt, September 5–13, 1994. Pathways. 1994;8 2:1–2. 12179680

[39] Greene M, Mehta M, Pulerwitz J, Wulf D, Bankole A, Singh S (2006). Involving men in reproductive health: Contributions to development. Men, Boys and Gender Equality.

[40] Matthews LT, Moore L, Milford C, Greener R, Mosery FN, Rifkin R (2015). "If I don't use a condom … I would be stressed in my heart that I've done something wrong": Routine prevention messages preclude safer conception counseling for HIV-infected men and women in South Africa. AIDS Behav.

[41] Matthews LT, Crankshaw T, Giddy J, Kaida A, Smit JA, Ware NC (2013). Reproductive decision-making and periconception practices among HIV-positive men and women attending HIV services in Durban, South Africa. AIDS Behav.

[42] Matthews LT, Smit JA, Moore L, Milford C, Greener R, Mosery FN (2015). Periconception HIV risk behavior among men and women reporting HIV-serodiscordant partners in KwaZulu-Natal, South Africa. AIDS Behav.

[43] Schwartz SR, West N, Phofa R, Yende N, Sanne I, Bassett J (2016). Acceptability and preferences for safer conception HIV prevention strategies: A qualitative study. Int J STD AIDS.

[44] Taylor TN, Mantell JE, Nywagi N, Cishe N, Cooper D (2013). 'He lacks his fatherhood': Safer conception technologies and the biological imperative for fatherhood among recently-diagnosed Xhosa-speaking men living with HIV in South Africa. Cult Health Sex.

[45] Khidir H, Psaros C, Greener L, O'Neil K, Mathenjwa M, Mosery FN (2017). Developing a safer conception intervention for men living with HIV in South Africa. AIDS Behav.

[46] Schwartz SR, Bassett J, Holmes CB, Yende N, Phofa R, Sanne I (2017). Client uptake of safer conception strategies: Implementation outcomes from the Sakh’umndeni safer conception clinic in South Africa. J Int AIDS Soc.

[47] Schwartz S, Davies N, Naidoo N, Pillay D, Makhoba N, Mullick S (2019). Clients’ experiences utilizing a safer conception service for HIV affected individuals: implications for differentiated care service delivery models. Reprod Health.

[48] Peltzer K, Jones D, Weiss SM, Shikwane E (2011). Promoting male involvement to improve PMTCT uptake and reduce antenatal HIV infection: A cluster randomized controlled trial protocol. BMC Public Health.

[49] Crankshaw TL, Matthews LT, Giddy J, Kaida A, Ware NC, Smit JA (2012). A conceptual framework for understanding HIV risk behavior in the context of supporting fertility goals among HIV-serodiscordant couples. Reprod Health Matters.

[50] Weber S, Zakaras JM, Hilliard S, Cohan D, Dworkin SL (2017). "Is it all right for me to have a baby or not?": Men living with HIV discuss fertility desires and interactions with providers. J Assoc Nurses AIDS Care.

[51] Nyblade L, Stockton MA, Giger K, Bond V, Ekstrand ML, Lean RM (2019). Stigma in health facilities: Why it matters and how we can change it. BMC Med.

[52] Wyrod R (2011). Masculinity and the persistence of AIDS stigma. Cult Health Sex.

[53] Mburu G, Ram M, Siu G, Bitira D, Skovdal M, Holland P (2014). Intersectionality of HIV stigma and masculinity in eastern Uganda: implications for involving men in HIV programmes. BMC Public Health.

[54] Dovel K, Dworkin SL, Cornell M, Coates TJ, Yeatman S (2020). Gendered health institutions: Examining the organization of health services and men’s use of HIV testing in Malawi. J Int AIDS Soc.

[55] Dovel K, Balakasi K, Gupta S, Mphande M, Robson I, Kalande P (2020). ’Missing men’ or missed opportunity? Men’s frequent use of health services in Malawi.

[56] Matthews LT, Bajunirwe F, Kastner J, Sanyu N, Akatukwasa C, Ng C (2016). "I always worry about what might happen ahead": Implementing safer conception services in the current environment of reproductive counseling for HIV-affected men and women in Uganda. Biomed Res Int.

[57] Matthews LT, Burns BF, Bajunirwe F, Kabakyenga J, Bwana M, Ng C (2017). Beyond HIV-serodiscordance: Partnership communication dynamics that affect engagement in safer conception care. PLoS ONE.

[58] Fransen-Dos Santos R, Guarinieri M (2017). Men living with HIV in serodiscordant relationships who desire a child/children. J Int AIDS Soc.

[59] Thummalachetty N, Mathur S, Mullinax M, DeCosta K, Nakyanjo N, Lutalo T (2017). Contraceptive knowledge, perceptions, and concerns among men in Uganda. BMC Public Health.

[60] Sharma M, Barnabas RV, Celum C (2017). Community-based strategies to strengthen men’s engagement in the HIV care cascade in sub-Saharan Africa. PLoS Med.

[61] Kiene SM, Gbenro O, Sileo KM, Lule H, Wanyenze RK (2017). How do we get partners to test for HIV?: predictors of uptake of partner HIV testing following individual outpatient provider initiated HIV testing in rural Uganda. AIDS Behav.

[62] Fleming PJ, Colvin C, Peacock D, Dworkin SL (2016). What role can gender-transformative programming for men play in increasing men’s HIV testing and engagement in HIV care and treatment in South Africa?. Cult Health Sex.

[63] Weiss SM, Zulu R, Jones DL, Redding CA, Cook R, Chitalu N (2015). The spear and shield intervention to increase the availability and acceptability of voluntary medical male circumcision in Zambia: a cluster randomised controlled trial. Lancet HIV.

[64] Stender SC, Rozario A (2020). "Khotla Bophelong Bo Botle": a gathering of men for health. J Int AIDS Soc.

[65] Wynn A, Rotheram-Borus MJ, Leibowitz AA, Weichle T, Roux IL, Tomlinson M (2017). Mentor mothers program improved child health outcomes at a relatively low cost in South Africa. Health Aff (Millwood).

[66] Khidir H, Mosery N, Greener R, Milford C, Bennett K, Kaida A (2020). Sexual relationship power and periconception HIV-risk behavior among HIV-infected men in serodifferent relationships. AIDS Behav.

[67] Bhatia DS, Harrison AD, Kubeka M, Milford C, Kaida A, Bajunirwe F (2017). The role of relationship dynamics and gender inequalities as barriers to HIV-serostatus disclosure: qualitative study among women and men living with HIV in Durban. South Afr Front Public Health.

[68] Rispel LC, Cloete A, Metcalf CA (2015). 'We keep her status to ourselves': Experiences of stigma and discrimination among HIV-discordant couples in South Africa. Tanzania Ukraine SAHARA J.

[69] Forrest JI, Kaida A, Dietrich J, Miller CL, Hogg RS, Gray G (2009). Perceptions of HIV and fertility among adolescents in Soweto, South Africa: Stigma and social barriers continue to hinder progress. AIDS Behav.

[70] Myer L, Morroni C, Cooper D (2006). Community attitudes towards sexual activity and childbearing by HIV-positive people in South Africa. AIDS Care.

[71] Chikovore J, Gillespie N, McGrath N, Orne-Gliemann J, Zuma T, Group ATS (2016). Men, masculinity, and engagement with treatment as prevention in KwaZulu-Natal. South Afr AIDS Care.

[72] Ministry of Health Lesotho, Centers for Disease Control and Prevention (CDC), University. IaC (2019). Lesotho population-based HIV impact assessment.

[73] Schwartz SR, Bassett J, Sanne I, Phofa R, Yende N, Van Rie A (2014). Implementation of a safer conception service for HIV-affected couples in South Africa. AIDS.

[74] Nakayiwa S, Abang B, Packel L, Lifshay J, Purcell DW, King R (2006). Desire for children and pregnancy risk behavior among HIV-infected men and women in Uganda. AIDS Behav.

[75] UNAIDS (2014). Fast-track: Ending the AIDS epidemic by 2030.

[76] Heffron R, Pintye J, Matthews LT, Weber S, Mugo N (2016). PrEP as peri-conception HIV prevention for women and men. Curr HIV/AIDS Rep.

[77] Matthews LT, Jaggernath M, Kriel Y, Smith PM, O'Neil K, Haberer JE (2019). Protocol for a longitudinal study to evaluate the use of tenofovir-based PrEP for safer conception and pregnancy among women in South Africa. BMJ Open.

[78] Adeyeye AO, Stirratt MJ, Burns DN (2018). Engaging men in HIV treatment and prevention. Lancet.

[79] Tomlinson M, Rotheram-Borus MJ, Swartz L, Tsai AC (2013). Scaling up mHealth: Where is the evidence?. PLoS Med.

[80] Kallander K, Tibenderana JK, Akpogheneta OJ, Strachan DL, Hill Z, ten Asbroek AH (2013). Mobile health (mHealth) approaches and lessons for increased performance and retention of community health workers in low- and middle-income countries: a review. J Med Internet Res.

[81] Fransen-Dos Santos R, Guarinieri M (2017). Men living with HIV in serodiscordant relationships who desire a child/children. J Int AIDS Soc.

